# Usefulness of a new foot switch for comport digestive endoscopic examination: a pilot study

**DOI:** 10.1007/s00464-025-11580-0

**Published:** 2025-02-24

**Authors:** Dong Seok Lee, Sang Gyun Kim, Byung-Wook Kim, Jeong-Seon Ji, Ji Yong Ahn

**Affiliations:** 1 Division of Gastroenterology, Department of Internal Medicine, Yeouido St. Mary’s Hospital, College of Medicine, The Catholic University of Korea, Seoul, Korea; 2 Department of Internal Medicine and Liver Research Institute, Seoul National University College of Medicine, Seoul, Korea; 3 Division of Gastroenterology, Department of Internal Medicine, Incheon St. Mary’s Hospital, College of Medicine, The Catholic University of Korea, Incheon, Korea; 4 Department of Gastroenterology, Asan Medical Center, University of Ulsan College of Medicine, Seoul, Korea

**Keywords:** Endoscopy, Foot switch, Comport, Stability, Pain, Musculoskeletal disorders

## Abstract

**Background:**

Foot switches are commonly used to record gastrointestinal lesions. However, prolonged use of foot switches can cause unstable posture, leading to musculoskeletal disorders. Therefore, this study aimed to develop and evaluate the usability of a compact foot switch for reducing musculoskeletal disorders among endoscopists.

**Methods:**

A new endoscopic foot switch was developed to reduce musculoskeletal disorders and was compared with a previous foot switch. Between January 1 and October 1, 2024, 50 expert endoscopists from five different centers analyzed its usability, postural stability, ability to reduce pain and work fatigue, and efficiency.

**Results:**

Compared with the conventional foot switch, the new foot switch showed favorable outcomes in terms of musculoskeletal disease-related factors, with better results in the following areas: comport endoscopic examination (6.5 [6–7] vs. 2 [1–2], p < 0.001), stable posture (6 [6–6] vs. 2 [2–3], p < 0.001), relief of musculoskeletal disorders (6 [5–6] vs. 3 [2–3], p < 0.001), efficiency of examination (6 [6–7] vs. 3 [2–3], p < 0.001), and applicability to other equipment (6 [6–7] vs. 2 [2–3], p < 0.001).

**Conclusions:**

We developed a new foot switch that can prevent musculoskeletal disorders among endoscopists. Further validation of its usefulness in various hospitals and users is required.

**Supplementary Information:**

The online version contains supplementary material available at 10.1007/s00464-025-11580-0.

Musculoskeletal disorders are important diseases in which damage to collagen connective tissue causes inflammation and pain. If not properly treated and prevented, they can lead to permanent injury and disability [1–3]. Many endoscopists are at risk for musculoskeletal disorders, and risk factors for musculoskeletal disorders include overuse injuries due to awkward postures, repetitive activities, and contact stress [4].

Many endoscopists experience musculoskeletal pain (39 − 89%) during endoscopic examinations, which is common in the neck and back [5–7]. These endoscopic activity-related injuries increase depending on age, work time, time per week, professional experience, and cumulative years of endoscopy [8, 9]. Given the complexity of these issues, there is an urgent need for targeted research on preventing musculoskeletal disorders among gastrointestinal endoscopists; however, such studies remain limited.

Inappropriate ergonomic settings can significantly increase the incidence of musculoskeletal disorders among high-volume endoscopists. In particular, asymmetric posture—characterized by an imbalance in the alignment of the limbs, spine, and head—is a critical factor contributing to musculoskeletal pain [10–12]. Prolonged asymmetric posture induces postural pressure, contributing to the aggravation of pain [13]. Over a long period, it can damage muscles, tendons, ligaments, bones, joints, and cartilage and lead to chronic disorders due to nerve compression or ischemia [14–16]. To prevent and treat musculoskeletal pain, maintaining “good” posture and “ideal” movement are important [17].

Foot switches are widely used in gastrointestinal endoscopic examinations and serve as the standard method for recording target lesions. Despite their utility, prolonged use of conventional foot switches can lead to asymmetric posture and musculoskeletal disorders (Fig. 1). While maintaining a symmetric posture is essential for good ergonomics during endoscopy, achieving this posture is challenging with conventional foot switches. Recently, the button method has been adopted in some hospitals to achieve good posture. This method involves using the endoscope’s built-in buttons to perform foot-switch functions, eliminating the need for a separate foot switch. Despite its advantages, the button method has several limitations. First, the preparation of additional modules is expensive. Second, the integration of an additional electrical module is only feasible with specialized endoscopic equipment. Third, prolonged use can cause pain in the finger joints [1]. To overcome these limitations, we designed a new foot switch using a manipulation method.

**Fig. 1:**
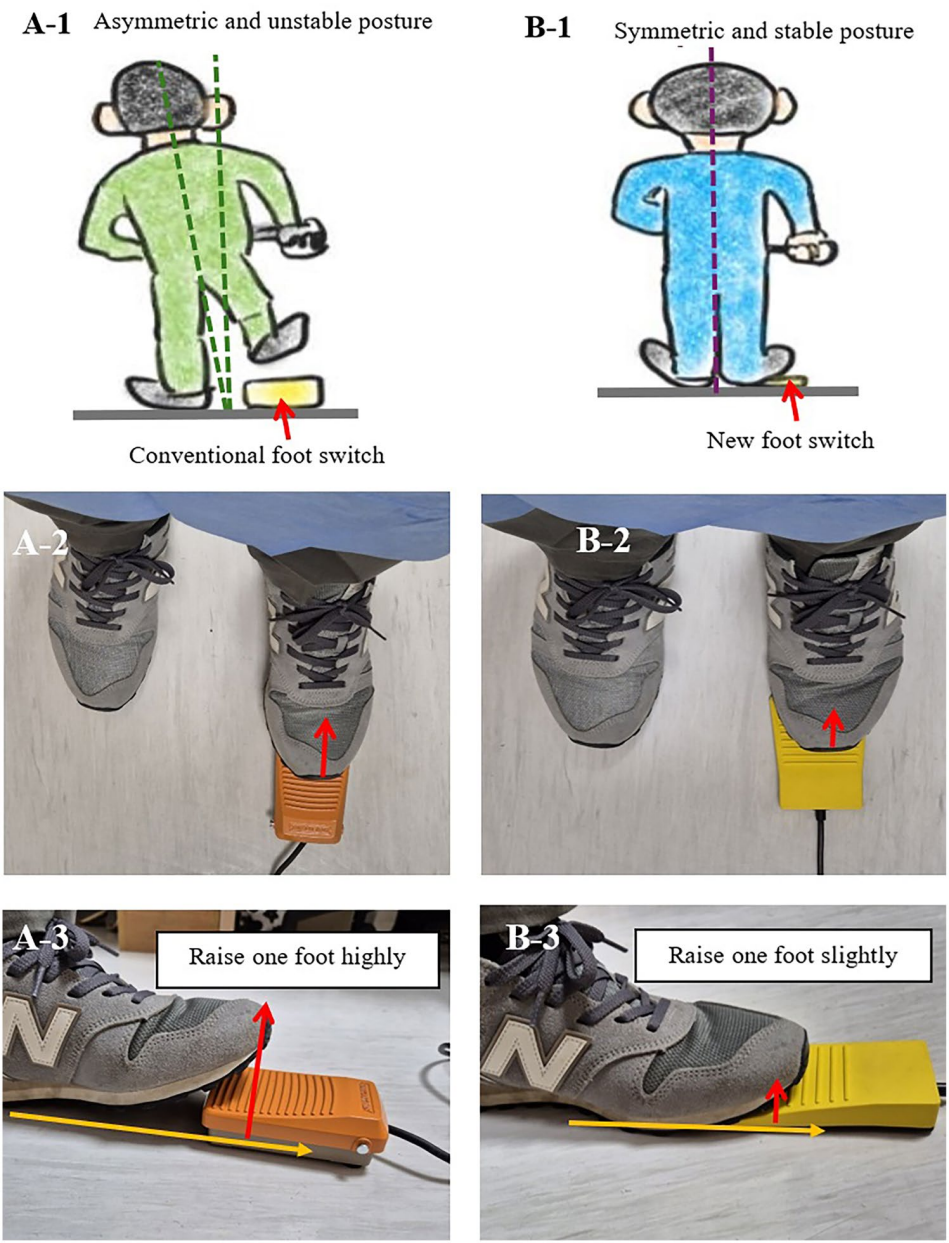
Comparison of postural stability while using the foot switches. **A** The examiner must lift one foot to work with the conventional foot switch. This causes an asymmetric unstable posture, and prolonged unstable posture causes back pain. **B** To operate the new foot switch, the examiner lifts only the front part of the foot. Compared to conventional foot switches, the examiner can maintain a symmetric, stable posture and feel comfortable

This study aimed to develop a new foot switch and evaluate its use in achieving stable posture and comfort, reducing musculoskeletal pain, and improving quality.

## Materials and methods

### Participants

Fifty endoscopists evaluated the new foot switch from January 1 to October 1, 2024, at Yeouido St. Mary’s Hospital, Incheon St. Mary’s Hospital, Seoul National University Hospital, Inha University Hospital, and Asan Medical Center, Seoul, Korea. Only expert members were selected as evaluators to eliminate as many subjective measurements as possible. These expert endoscopists performed more than 1000 esophagogastroduodenoscopies and 500 colonoscopies. This study was conducted in accordance with the principles of the Declaration of Helsinki. This study was approved by the Ethics Committee of Incheon St. Mary’s Hospital, Catholic University of Korea (IRB number: OC24EASI0097).

### Conventional foot switch

**Fig. 2:**
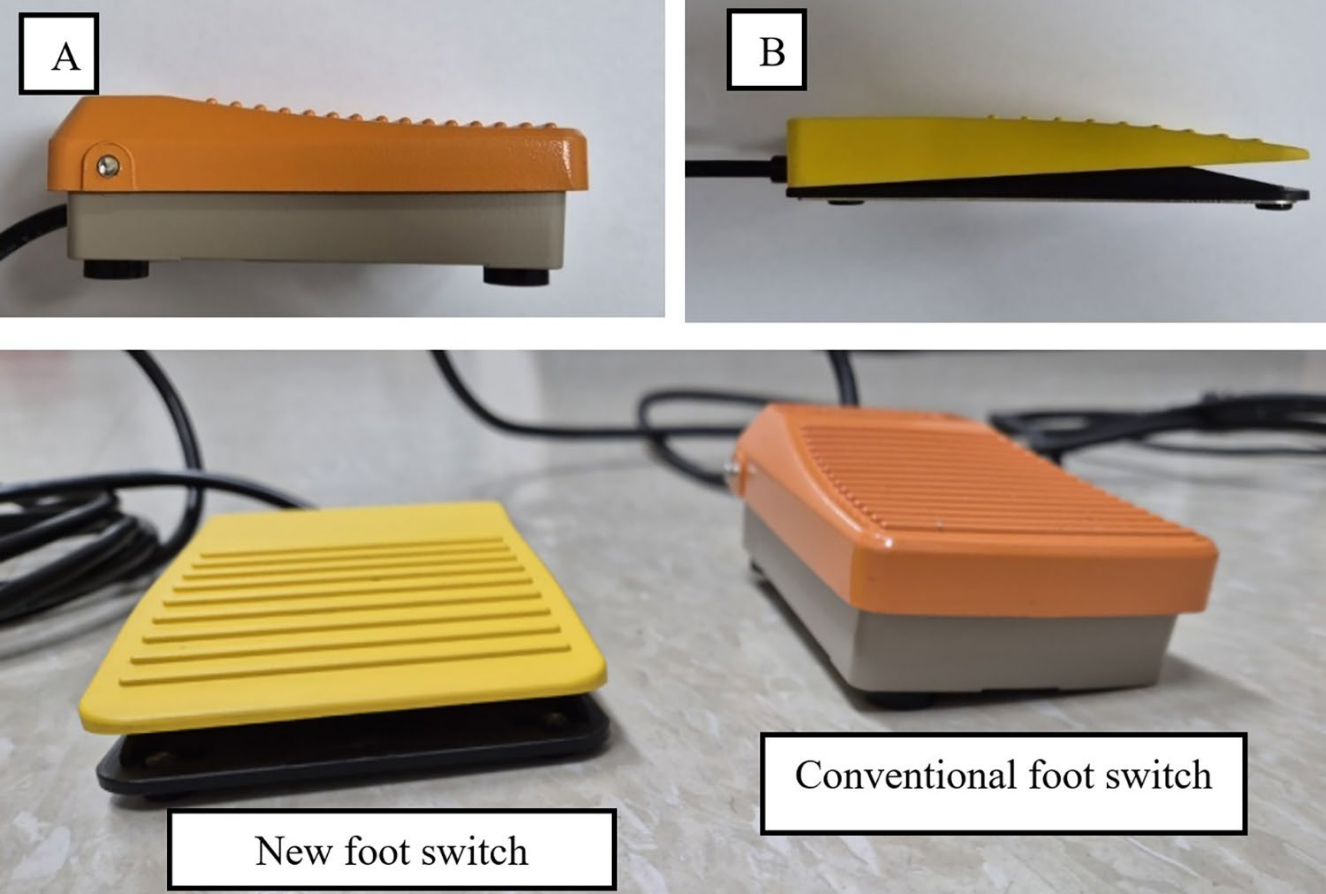
Designs of two-foot switches: **A** Conventional foot switch. **B** New foot switch. The new foot switch was designed to be low in height, allowing users to maintain a stable posture. When the operator continues to step on the foot switch, the switch does not operate. It operates when the operator quickly steps up and down on a foot-switch

Currently, several foot switches are used for digestive endoscopic examinations. We selected a conventional foot switch (Foot-switch; Hando, Korea) that is commonly used in Korea for comparison in this study. The outer dimensions of the product were 11.4 × 7.2 × 2.7 cm, and the vertical working length was 8 mm (Fig. 2). When the foot switch was not pressed, the function of the switch was turned off. When the foot switch was not pressed, its function remained inactive. Conversely, pressing the foot switch activated the device. As a result, the examiner had to consistently raise one leg by more than 5 cm to prevent unintentional activation of the switch (Fig. 1).

### Newly developed foot switch

An experienced endoscopist at Yeouido St. Mary’s Hospital has developed a new foot switch. The newly designed foot switch is illustrated in Fig. 2. The new foot switch measures 11 × 7 × 1.5 cm and was constructed at minimum height to minimize postural imbalance. This new foot switch can help reduce the incidence of musculoskeletal disorders. This product was developed and manufactured for research purposes. The patent number for the product is 10-2024-00992042 (Comport foot-switch; KAYEON, Korea). The author declares no conflict of interest with the company associated with the patented device discussed in this study. First, it enabled the examiner to stand in a comfortable posture. To capture a snapshot, the examiner moved only part of the front foot slightly upward and downward. The examiner did not need to lift their legs or place both feet on the floor. Second, the examiner stepped on the foot switch and there was no need to try to locate the foot switch (Fig. 1).

### Outcome measures

The primary outcome was the degree of comfort when using the foot switch for endoscopic examination, which was evaluated by endoscopists from different hospitals. Before using the new foot switch, the participants were fully trained in the manipulation method of the new foot switch. After the training period, the participants evaluated the foot switch for more than 1 month during gastroscopy and colonoscopy. The participants used a score table with a 7-point Likert scale for evaluation. The new questionnaire included a critical factor for evaluating musculoskeletal disorders associated with foot-switch usage and endoscopy. The 7-point Likert scale included the following options: strongly disagree, disagree, somewhat disagree, agree or disagree, somewhat agree, agree, and strongly disagree (1 = strongly disagree, 2 = disagree, 3 = somewhat disagree, 4 = agree or disagree, 5 = somewhat agree, 6 = agree, and 7 = strongly agree). The scores covered the following topics: (1) comport manipulation of the foot switch, (2) comport endoscopic examination and stable posture of the examiner, (3) musculoskeletal pain (prevention and relief), (4) overall fatigue reduction, (5) efficiency of endoscopic examination, and (6) applicability to other equipment (Table 1). “Comport manipulation of the foot switch” indicates how comfortable manipulation of the foot switch is. “Comport in the endoscopic exam” refers to the comfort of performing an endoscopic examination when using a foot switch. Musculoskeletal pain prevention and relief refer to the degree of pain prevention and relief in each area. “Overall fatigue reduction” assesses the reduction in the examiner’s fatigue. “Applicability to other equipment” indicates whether the foot switch can be applied to other endoscopic and surgical devices.

**Table 1:** Suitability of the foot switch for comport endoscopy using a 7-point Likert scale

Index	New foot switch	Control foot switch	*p*-value
Comport manipulation of the foot switch	6.5 [6, 7]	2 [1, 2]	< 0.001
Comport endoscopic exam	6 [6, 7]	2 [2–2]	< 0.001
Stable posture of the examiner	6 [6–6]	2 [2, 3]	< 0.001
Musculoskeletal pain (prevention and relief)			
Neck pain (prevention and reduction)	6 [5, 6]	3 [2, 3]	< 0.001
Back pain (prevention and reduction)	6.5 [6, 7]	2.5 [2, 3]	< 0.001
Shoulder pain (prevention and reduction)	6 [6, 7]	3 [3, 4]	< 0.001
Pelvic pain (prevention and reduction)	6 [6, 7]	2 [2, 3]	< 0.001
Knee pain (prevention and reduction)	6 [6, 7]	2 [2, 3]	< 0.001
Overall fatigue reduction	6 [6, 7]	2 [2, 3]	< 0.001
Efficiency of endoscopic examination			
Inspection time	6 [6, 7]	2 [2, 3]	< 0.001
Concentration	6 [5.25–6]	2 [1.5–2]	< 0.001
Applicability with other equipment			
Endoscopic mucosal resection	6 [6, 7]	2 [2, 3]	< 0.001
Endoscopic submucosal dissection	6 [6, 7]	2 [2, 3]	< 0.001
Endoscopic retrograde cholangiopancreatography	6 [6–6]	2 [2–2]	< 0.001
Surgical knife such as Bovie knife	6 [6–6.75]	2 [2–2.75]	< 0.001

Evaluation scores were measured by an expert group. Likert scale (1 = strongly disagree, 2 = disagree, 3 = somewhat disagree, 4 = agree or disagree, 5 = somewhat agree, 6 = agree, 7 = strongly agree). Values are presented as median (interquartile range). Likert scale (1 = strongly disagree, 2 = disagree, 3 = somewhat disagree, 4 = agree or disagree, 5 = somewhat agree, 6 = agree, 7 = strongly agree)

### Details of the device setting

To evaluate each foot switch, a standard single-channel endoscope (GIF-H290 and CF-H290; Olympus) connected to the USB connector of an endoscopic equipment computer was used.

### Statistical analyses

Statistical analyses were performed using Rex version 3.0.3 (Rex Soft Inc., Seoul, Korea). Continuous variables are reported as medians with interquartile ranges. T-tests were performed to compare continuous variables between the two groups, and a two-tailed p-value < 0.05 was considered statistically significant.

## Results

The participants compared various musculoskeletal disease-related factors of the two (new and existing) foot switches (Figs. 1 and 2 and Table 1). No malfunction in electrical function was observed during the evaluation. The snapshot recordings were satisfactory for both foot switches. However, compared to the conventional foot switch, the new foot switch showed better results for comport manipulation of the foot switch (6.5 [6, 7] vs. 2 [1, 2], p < 0.001), comport endoscopic exam (6.5 [6, 7] vs. 2 [2], p < 0.001), stable posture (6 [6] vs. 2 [2, 3], p < 0.001), pain relief (6 [5, 6] vs. 3 [2, 3], p < 0.001), overall fatigue reduction (6 [6, 7] vs. 2 [2, 3], p < 0.001), efficiency of endoscopic examination (6 [6, 7] vs. 3 [2, 3], p < 0.001), and applicability to other equipment (6 [6, 7] vs. 2 [2, 3], p < 0.001).

## Discussion

Gastroenterologists are exposed to high-intensity musculoskeletal disorders [1, 18], which are important issues regarding workers’ health and the quality of the examinations performed. Previous studies have focused on the prevalence of musculoskeletal injuries and have assessed the role of ergonomics among practicing gastroenterologists [19]. These studies examined strategies to minimize the risk of endoscopy-related injuries, optimize the well-being of endoscopists, and maximize overall system performance. Ergonomics in endoscopy can be categorized into three main areas: cognitive skills, technical skills, and non-technical skills [20]. Cognitive skills involve understanding techniques to minimize musculoskeletal strain during endoscopy, such as ergonomic education and training modules focusing on injury prevention. Technical skills involve appropriate body positioning, posture, and workstation adjustments, which can significantly reduce physical stress. While the novel endoscopic foot-switch in this study offers a promising supplement to technical skills, other methods, such as height-adjustable workstations, ergonomic chairs, and targeted physical exercises, are also effective in mitigating musculoskeletal strain. Finally, non-technical skills encompass environmental factors external to the endoscopist, such as team member interactions, patient positioning, and ensuring adequate rest periods.

A foot switch was used to record lesions during gastrointestinal endoscopy. Various foot switches have been introduced and are commonly used. However, prolonged use can cause musculoskeletal disorders. We developed a foot switch that can help endoscopists perform examinations comfortably in the standing position. The gastrointestinal endoscopy specialists who participated in our study affirmed the potential of the foot switch to facilitate a comfortable endoscopic examination.

To our knowledge, this study is the first to address foot switching as a crucial risk factor for musculoskeletal health disorders among Korean GI endoscopists. A new foot switch is required for ergonomic device optimization in GI endoscopy.

The advantages of the new foot switch are as follows: First, it is compatible with existing endoscopy equipment. Second, it can help reduce musculoskeletal disorders in the standing position. Third, it improves the efficiency and quality of gastrointestinal endoscopy. Fourth, it can be used at various foot-switch sites. Fifth, foot switches can be used in other for endoscopic techniques such as endoscopic mucosal resection, endoscopic submucosal dissection, endoscopic retrograde cholangiopancreatography, and surgical knives such as the Bovie knife.

This study had some limitations. First, the evaluation could be biased and subjective because the scoring system was constructed with a 7-point Likert scale Second, selection bias may have been present because of the small number of participants. In the future, it would be beneficial to conduct additional multicenter studies.

In conclusion, we developed and evaluated a new foot switch that can reduce musculoskeletal disorders. The overall performance was superior to that of a conventional foot switch. We believe that this new foot switch will help reduce and protect endoscopists from musculoskeletal diseases and ultimately improve their personal health and work performance.

## Supplementary Information

Below is the link to the electronic supplementary material.

Supplementary file1 (MP4 2776 KB)

Supplementary file2 (PDF 51 KB)
