# Supplementary material for: Usefulness of a new foot switch for comport digestive endoscopic examination: a pilot study
Source: Surg Endosc. 2025 Feb 24;39(4):2458–63. doi: 10.1007/s00464-025-11580-0 (PMC11933142; doi:10.1007/s00464-025-11580-0)
Supplement: Supplementary file 2 — Supplementary file2 (PDF 51 KB) [file 464_2025_11580_MOESM2_ESM.pdf]

Foot switch evaluation sheet

This questionnaire evaluated the usefulness of a new foot switch for comport digestive endoscopic activity.

Previous endoscopic examination experience

- Total number of prior esophagogastroduodenoscopies: _______
- Total number of prior colonoscopies: ________

Choose the foot switch type.

- Conventional foot switch ☐
- New foot switch ☐

Please read the questions below and place a checkmark (☑) in the box at the bottom of the relevant item.

1. Comport manipulation of the foot switch

To take a snapshot, the examiner moves only part of the front foot slightly upward and downward. The examiner does not need to lift their legs or place both feet on the floor.

1	2	3	4	5	6	7
Strongly Disagree	Disagree	Somewhat disagree	Agree or disagree	Somewhat agree	Agree	Strongly agree
☐	☐	☐	☐	☐	☐	☐

2. Comport endoscopic exam

Despite its long-term use, this foot switch does not induce asymmetric posture and musculoskeletal disorders. (Asymmetric posture, which involves an imbalance of the limbs, spine, and head, is a crucial factor contributing to musculoskeletal pain).

1	2	3	4	5	6	7

Strongly Disagree	Disagree	Somewhat disagree	Agree or disagree	Somewhat agree	Agree	Strongly agree
☐	☐	☐	☐	☐	☐	☐

3. Stable posture of the examiner

This foot switch helps stabilize the endoscopist’s posture. (Symmetric posture induces comport endoscopic exam by the good posture during endoscopy).

1	2	3	4	5	6	7
Strongly Disagree	Disagree	Somewhat disagree	Agree or disagree	Somewhat agree	Agree	Strongly agree
☐	☐	☐	☐	☐	☐	☐

4. Musculoskeletal pain relief (Neck pain prevention and reduction):

This foot switch is helpful for musculoskeletal pain relief and prevention in the neck area.

1	2	3	4	5	6	7
Strongly Disagree	Disagree	Somewhat disagree	Agree or disagree	Somewhat agree	Agree	Strongly agree
☐	☐	☐	☐	☐	☐	☐

5. Musculoskeletal pain relief (Back pain prevention and reduction)

This foot switch is helpful for musculoskeletal pain relief and prevention in the back area.

1	2	3	4	5	6	7
Strongly Disagree	Disagree	Somewhat disagree	Agree or disagree	Somewhat agree	Agree	Strongly agree
☐	☐	☐	☐	☐	☐	☐

6. Musculoskeletal pain relief (Shoulder pain prevention and reduction)

This foot switch is helpful for musculoskeletal pain relief and prevention in the shoulder area.

1	2	3	4	5	6	7
Strongly Disagree	Disagree	Somewhat disagree	Agree or disagree	Somewhat agree	Agree	Strongly agree
☐	☐	☐	☐	☐	☐	☐

7. Musculoskeletal pain relief (Pelvic pain prevention and reduction)

This foot switch is helpful for musculoskeletal pain relief and prevention in

the pelvic area.

1	2	3	4	5	6	7
Strongly Disagree	Disagree	Somewhat disagree	Agree or disagree	Somewhat agree	Agree	Strongly agree
☐	☐	☐	☐	☐	☐	☐

8. Musculoskeletal pain relief (knee pain prevention and reduction)

This type of foot switch is helpful for musculoskeletal pain relief and prevention in the knee area.

1	2	3	4	5	6	7
Strongly Disagree	Disagree	Somewhat disagree	Agree or disagree	Somewhat agree	Agree	Strongly agree
☐	☐	☐	☐	☐	☐	☐

9. Overall fatigue reduction

- This foot switch reduces overall fatigue associated with endoscopic activity.
- This foot switch reduces inappropriate ergonomic settings, which increases musculoskeletal disorders among high-volume endoscopists.

1	2	3	4	5	6	7
Strongly Disagree	Disagree	Somewhat disagree	Agree or disagree	Somewhat agree	Agree	Strongly agree
☐	☐	☐	☐	☐	☐	☐

10. Inspection time (Efficiency of endoscopic examination)

This foot switch helps shorten endoscopy time (regarding manipulation and musculoskeletal pain).

1	2	3	4	5	6	7
Strongly Disagree	Disagree	Somewhat disagree	Agree or disagree	Somewhat agree	Agree	Strongly agree
☐	☐	☐	☐	☐	☐	☐

11. Concentration (Efficiency of endoscopic examination:)

This foot switch helps concentration (regarding manipulation and

musculoskeletal pain).

1	2	3	4	5	6	7
Strongly Disagree	Disagree	Somewhat disagree	Agree or disagree	Somewhat agree	Agree	Strongly agree
☐	☐	☐	☐	☐	☐	☐

12. Applicability with endoscopic mucosal resection

This foot switch can be applied for comfort endoscopic mucosal resection.

1	2	3	4	5	6	7
Strongly Disagree	Disagree	Somewhat disagree	Agree or disagree	Somewhat agree	Agree	Strongly agree
☐	☐	☐	☐	☐	☐	☐

13. Applicability with endoscopic submucosal dissection

This foot switch can be applied for comfort endoscopic submucosal dissection.

1	2	3	4	5	6	7
Strongly Disagree	Disagree	Somewhat disagree	Agree or disagree	Somewhat agree	Agree	Strongly agree
☐	☐	☐	☐	☐	☐	☐

14. Applicability with endoscopic retrograde cholangiopancreatography

This foot switch can be applied for comfort endoscopic retrograde cholangiopancreatography.

1	2	3	4	5	6	7
Strongly Disagree	Disagree	Somewhat disagree	Agree or disagree	Somewhat agree	Agree	Strongly agree
☐	☐	☐	☐	☐	☐	☐

15. Applicability with surgical knife such as Bovie knife

The foot switch can be applied for the comfort use of a surgical knife such as Bovie knife.

1	2	3	4	5	6	7
Strongly Disagree	Disagree	Somewhat disagree	Agree or disagree	Somewhat agree	Agree	Strongly agree
☐	☐	☐	☐	☐	☐	☐
